# Geographic distribution of the V1016G knockdown resistance mutation in *Aedes albopictus*: a warning bell for Europe

**DOI:** 10.1186/s13071-022-05407-3

**Published:** 2022-08-05

**Authors:** Verena Pichler, Beniamino Caputo, Vera Valadas, Martina Micocci, Cintia Horvath, Chiara Virgillito, Mustafa Akiner, Georgios Balatsos, Christelle Bender, Gilles Besnard, Daniel Bravo-Barriga, Rubén Bueno-Mari, Francisco Collantes, Sarah Delacour-Estrella, Enkelejda Dikolli, Elena Falcuta, Eleonora Flacio, Ana L. García-Pérez, Katja Kalan, Mihaela Kavran, Gregory L’Ambert, Riccardo P. Lia, Eduardo Marabuto, Raquel Medialdea, Rosario Melero-Alcibar, Antonios Michaelakis, Andrei Mihalca, Ognyan Mikov, Miguel A. Miranda, Pie Müller, Domenico Otranto, Igor Pajovic, Dusan Petric, Maria Teresa Rebelo, Vincent Robert, Elton Rogozi, Ana Tello, Toni Zitko, Francis Schaffner, Joao Pinto, Alessandra della Torre

**Affiliations:** 1 Dipartimento di Sanità Pubblica & Malattie Infettive, Università di Roma Sapienza, Rome, Italy; 2 Global Health and Tropical Medicine, Instituto De Higiene E Medicina Tropical, Universidade Nova De Lisboa, Lisbon, Portugal; 3 University of Agricultural Sciences and Veterinary Medicine, Cluj-Napoca, Romania; 4 Recep Tayyip Erdoğan Üniversitesi, Rize, Turkey; 5 Laboratory of Insects & Parasites of Medical Importance, Benaki Phytopathological Institute, Kifisia, Greece; 6 Syndicat de Lutte Contre Les Moustiques du Bas-Rhin, Strasbourg, France; 7 Entente Interdépartementale Rhône-Alpes pour la Démoustication, Chindrieux, France; 8 Animal Health Department, Veterinary Faculty, University of Extremadura (UEx), Cáceres, Spain; 9 Lokimica Laboratorios, Valencia, Spain; 10 University of Murcia, Murcia, Spain; 11 University of Zaragoza, Saragossa, Spain; 12 Institute of Public Health, Tiranë, Albania; 13 Cantacuzino, National Military-Medical Institute of Research and Development, Bucharest, Romania; 14 University of Applied Sciences of Southern Switzerland, Manno, Switzerland; 15 Neiker-Basque Institute for Agricultural Research and Development, Derio, Spain; 16 University of Primorska, Koper, Slovenia; 17 University of Novi Sad, Novi Sad, Serbia; 18 Università degli Studi di Bari Aldo Moro, Bari, Italy; 19 Museum of Zoology, Senckenberg Natural History Collections Dresden, Dresden, Germany; 20 Ministry for Health of Malta, Valetta, Malta; 21 Facultad de Ciencias Biológicas, Universidad Complutense de Madrid, Madrid, Spain; 22 National Centre of Infectious and Parasitic Diseases, Sofia, Bulgaria; 23 Applied Zoology and Animal Conservation, University of the Balearic Islands, Palma, Spain; 24 Swiss Tropical and Public Health Institute, Allschwil, Switzerland; 25 University of Basel, Basel, Switzerland; 26 University of Montenegro, Podgorica, Montenegro; 27 CESAM-Ciências, Faculdade de Ciências da Universidade de Lisboa, , Lisbon, Portugal; 28 Mivegec Laboratory, Institut de Recherche pour le Développement, Centre National de la Recherche Scientifique, University of Montpellier, Montpellier, France; 29 Institute of Public Health of Split-Dalmatia County, Split, Croatia; 30 Francis Schaffner Consultancy, Riehen, Switzerland

**Keywords:** Mosquito, *Aedes albopictus*, Insecticide resistance, Kdr, Europe, Integrated vector management, Arbovirus vector, Vector control

## Abstract

**Background:**

Colonization of large part of Europe by the Asian tiger mosquito *Aedes albopictus* is causing autochthonous transmission of chikungunya and dengue exotic arboviruses. While pyrethroids are recommended only to reduce/limit transmission, they are widely implemented to reduce biting nuisance and to control agricultural pests, increasing the risk of insurgence of resistance mechanisms. Worryingly, pyrethroid resistance (with mortality < 70%) was recently reported in *Ae. albopictus* populations from Italy and Spain and associated with the V1016G point mutation in the voltage-sensitive sodium channel gene conferring knockdown resistance (*kdr*). Genotyping pyrethroid resistance-associated *kdr* mutations in field mosquito samples represents a powerful approach to detect early signs of resistance without the need for carrying out phenotypic bioassays which require availability of live mosquitoes, dedicated facilities and appropriate expertise.

**Methods:**

Here we report results on the PCR-genotyping of the V1016G mutation in 2530 *Ae. albopictus* specimens from 69 sampling sites in 19 European countries.

**Results:**

The mutation was identified in 12 sites from nine countries (with allele frequencies ranging from 1 to 8%), mostly distributed in two geographical clusters. The western cluster includes Mediterranean coastal sites from Italy, France and Malta as well as single sites from both Spain and Switzerland. The eastern cluster includes sites on both sides of the Black Sea in Bulgaria, Turkey and Georgia as well as one site from Romania. These results are consistent with genomic data showing high connectivity and close genetic relationship among West European populations and a major barrier to gene flow between West European and Balkan populations.

**Conclusions:**

The results of this first effort to map *kdr* mutations in *Ae. albopictus* on a continental scale show a widespread presence of the V1016G allele in Europe, although at lower frequencies than those previously reported from Italy. This represents a wake-up call for mosquito surveillance programs in Europe to include PCR-genotyping of pyrethroid resistance alleles, as well as phenotypic resistance assessments, in their routine activities.

**Graphical Abstract:**

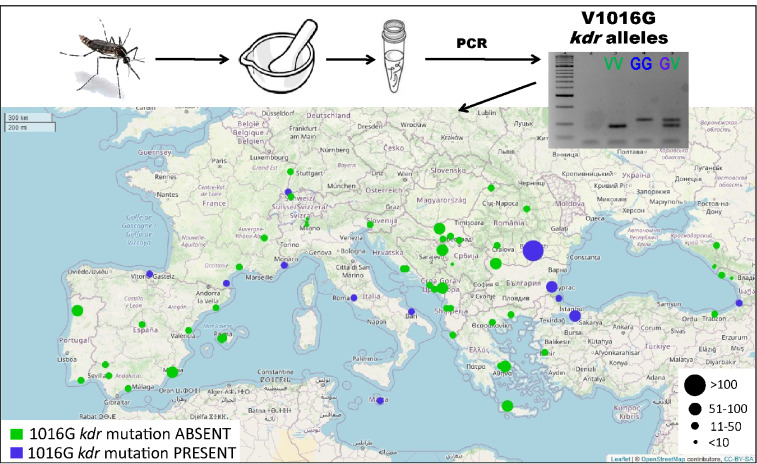

**Supplementary Information:**

The online version contains supplementary material available at 10.1186/s13071-022-05407-3.

## Background

In the last few decades, mosquito-borne arboviruses (i.e. arthropod-borne viruses, such as dengue and chikungunya) have undergone an extraordinary spread as a consequence of the colonization of large tropical and temperate regions by invasive *Aedes* mosquito species [1, 2]. In particular, in less than 40 years, *Aedes albopictus* has invaded all continents except Antarctica—thanks to the capacity of its eggs to sustain both dessication and low temperatures—and has become an increasing public health concern also in temperate regions. In fact, several autochthonous outbreaks of dengue (dengue virus [DENV] in Croatia, France, Spain and Italy [3–5]) and two major chikungunya (chikungunya virus [CHIKV]) outbreaks in Italy [6, 7] have been reported since the species’ appearance in Europe. Since no specific medical treatment exists for these diseases, integrated vector management is the only available strategy to limit the public health burden [8].

To reduce mosquito nuisance and the risk of disease outbreaks, European guidelines for the surveillance of invasive mosquitoes [9] recommend larval source reduction and larvicide applications. In contrast, pyrethroid-based adulticidal interventions are recommended only in the cases of ongoing—or high risk of—virus transmission, when a fast and effective abatement of adult mosquitoes is necessary. Pyrethroids, which are the only insecticide class for mosquito adulticide spraying registered in Europe [10, 11], interact with the voltage-sensitive sodium channel (VSSC) and interfere with the transmission of nervous signals, resulting in fast knockdown and eventually death of the mosquito [12]. However, their effectiveness is increasingly compromised by the rise of insecticide resistance. This is observed in all major mosquito vector species, including *Aedes aegypti* and Afrotropical malaria vectors, and has been recently reported in *Ae. albopictus* populations from both the native [13–20] and the invasive ranges [21–26], including populations in Italy and Spain [27–29].

Target site mutations in the *vssc* gene, conferring knockdown resistance (*kdr*), are among the best-characterized mechanisms contributing to pyrethroid resistance across all major mosquito vector species. These *kdr* mutations weaken the binding of the pyrethroid insecticide to the sodium channel, thereby reducing the knockdown effect [12]. Studies on *Ae. aegypti* have identified several point mutations (reviewed by Moyes et al. [30]) in the S6 transmembrane segments of domain II and III of the VSSC protein that constitute the pyrethroid binding site [31]. Among these, V410L, S989P, I1011M, V1016G and F1534C show the strongest association with resistance phenotypes [30]. Moreover, several mutations act synergistically, resulting in enhanced levels of resistance. In particular, functional assays showed that, compared to the wild type, the co-occurrence of the three mutations S989P, V1016G and F1534C decreases the susceptibility to permethrin and deltamethrin by 1100- and 90-fold, respectively [32].

Despite studies having so far mostly focused on major vector species, in the last decade a few mutations within the *vssc* gene have also been identified in *Ae. albopictus*, in particular in positions 1534 (F1534C/S/L/W/R; [19, 33–36]), 1016 (V1016G/I [34, 36, 37]) and 1532 (I1532T [38]). Among these, the only alleles confirmed to be associated with strong pyrethroid resistance phenotypes are 1534C [37], 1534S [18, 36, 37, 39] and 1016G [29, 37]. The latter has been shown to confer the highest levels of resistance to different pyrethroids [32, 37] and has been reported from the species native range [34, 36, 37], as well as from Reunion Island in the Indian Ocean [34]. In the European region, it has only been detected in Italy, where it is widespread and reaches alarming frequencies of up to 45% in some coastal sites [29, 40].

Genotyping pyrethroid resistance-associated mutations in mosquito samples from natural populations represents a powerful approach to detect early signs of resistance without the need of carrying out phenotypic bioassays that require availability of live mosquitoes, dedicated facilities and appropriate expertise [41]. Indeed, PCR-based approaches have proved to be instrumental in monitoring the onset and spread of *kdr* alleles and in raising awareness of insecticide resistance in *Ae. aegypti* and major malaria vectors [41].

The aim of this work was to map the presence and frequency of the V1016G mutation in *Ae. albopictus* populations across Europe.

## Methods

European *Ae. albopictus* specimens were sampled between 2015 and 2020 within the framework of AIM-COST Action (http://www.aedescost.eu) and of the ARBOMONITOR projects. Mosquitoes were collected by either ovitraps, larval sampling or adult trapping methods (see Additional file 1: Table S1). Larvae collected in the field or hatched from ovitrap-collected eggs were reared to adults under standard insectary conditions.

The DNA was extracted from single legs or whole individual mosquito carcasses using the DNAzol® [42] or CTAB [43] methods. The allele-specific PCR (AS-PCR) assay for V1016G genotyping was performed either on DNA extracted from single specimens or on pooled DNA extracted from three specimens [40]. In the case of detection of the 1016G allele in one of the pools, genotyping by PCR of each of the three specimens was performed separately.

For a subset of specimens, a fragment of domain II of the *vssc* gene was sequenced following the protocol described by Kasai et al. [37]. This comprised all specimens identified as either homozygotes or heterozygotes for the mutated 1016G allele by AS-PCR, as well as a subset of randomly chosen homozygotes for the susceptible 1016V allele from each country. PCR products were purified using the SureClean Kit (Bioline; Meridian Bioscience, Cincinnati, OH, USA), and the amplicons were sequenced either at BMR Genomics s.r.l. (Padua, Italy) or at STAB Vida (Oeiras, Portugal). Results from sequencing and AS-PCR genotyping were compared and the accuracy of the AS-PCR was estimated as the number of correct assessments divided by the total number of observations, taking the DNA sequencing results as the gold standard. Genotyping results were deposited in VectorBase.org (Project ID: VBP0000793). An interactive map reporting frequencies of the V1016G mutation per site (including also reports from previous publications; [29, 40]) was created using the Leaflet package for R (https://rstudio.github.io/leaflet/) in R studio version 2019. The database was better visualized by exploiting “tydiverse” and “ddply.” Finally, the “classInt” package was used to obtain the scales of frequency of V1016G. The interactive map code and the data are available at https://randomxsk8.github.io/MedEnt_Sapienza/resist_map.html.

## Results and discussion

Here we report for the first time the presence of the V1016G mutation in European *Ae. albopictus* populations outside Italy. Overall, 2530 specimens from 69 sampling sites in 19 European countries were PCR-genotyped (Additional file 1:Table S1). For a subsample of 265 specimens, a fragment of domain II of the *vssc* gene, including position 1016, was also sequenced to validate the PCR results. Consistently with the results reported by Pichler et al. [40], the AS-PCR assay accuracy was 94%. All mismatches (*N* = 16) between AS-PCR and sequencing were due to specimens homozygous for the 1016V susceptible allele and PCR-genotyped as heterozygotes (Table 1). This result confirms the previously reported slight overestimation of mutant allele detection by AS-PCR [40] and highlights the relevance of confirming PCR results by sequencing individuals carrying the resistant 1016G allele, particularly when the allele is detected for the first time in a region. Sequence analysis of the 16 incorrectly PCR-genotyped specimens did not reveal mutations in primer binding sites. Since 12 out of the 16 specimens came from only three sampling sites (i.e. Burgas in Bulgaria, Bucharest in Romania and Basauri in Spain), it is possible to hypothesize that low-quality DNA may have biased the PCR reaction.

**Table 1:** Comparison of genotyping results obtained by sequencing the V1016G knockdown resistance mutation of the *vssc* gene in *Aedes albopictus* and by allele-specific-PCR assay

Genotyping results by AS-PCR assay	Genotyping results by sequencing			Total
	VV	VG	GG	
VV	203	–	–	203
VG	*16*	45	–	61
GG	–	–	1	2
Total	219	45	1	265

Discordances are shown in italics/underlined

*AS-PCR* Allele-specific PCR, *V* 1016V wild-type allele,* G* 1016G knockdown resistance (*kdr*) allele

Noteworthy, the amino acid valine at position 1016 of the VSSC protein was encoded by the GTG codon instead of the wild-type GTA codon in five specimens, including two heterozygote GTA/GTG specimens, one homozygote GTG/GTG specimen from Greece and two heterozygote GTA/GTG specimens from Serbia. This synonymous substitution has already been observed in Italian specimens [40] and shown to have no impact on AS-PCR results. Moreover, as already described by Zhou et al. [36] and Pichler et al. [40], the amplicon lengths varied by about 10 bp. This variation is due to insertions present in the intron 20 of the *vssc* gene, abutting codon position 1016. However, this does not interfere with the correct identification of the 1016V and 1016G alleles.

**Fig. 1:**
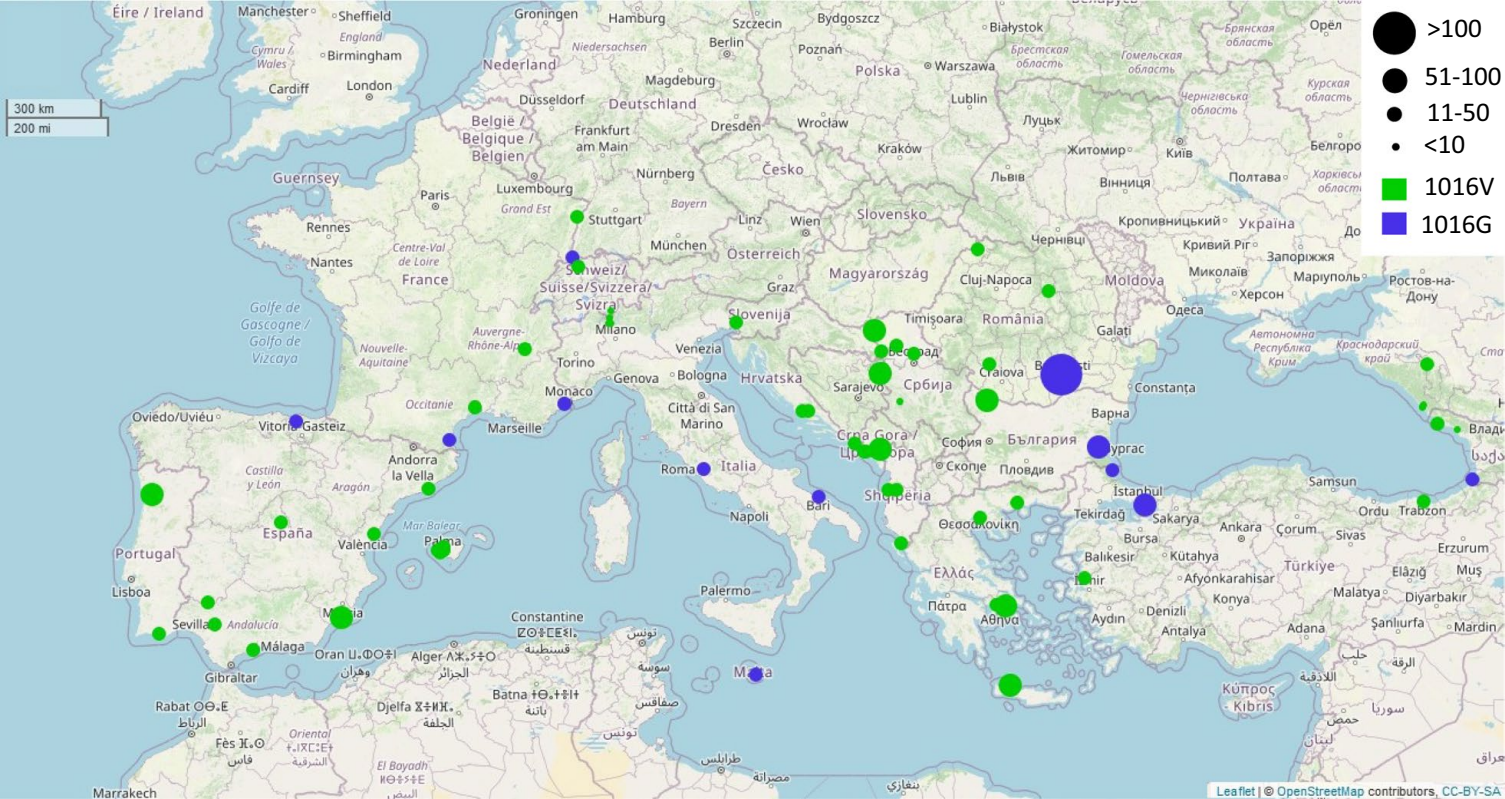
Distribution of the V1016V knockdown locus (*kdr*) in the gene encoding the voltage-sensitive sodium channel (*vssc*) in *Aedes albopictus* across Europe. Each dot represents a sampling site, while the size corresponds to the number of specimens that were PCR-genotyped for the V1016V *kdr* locus from that site. Details on the sampling sites and the 1016G allele frequencies per site are given in Additional file 1: Table S1. Green dots represent samples with wild-type 1016V allele only; blue dots represent samples where the *kdr* 1016G allele was detected. The georeferenced map was produced using the leaflet package (https://rstudio.github.io/leaflet/) in RStudio 4.1.2 with map data from OpenStreetMap contributor

The combined AS-PCR and sequencing results reveal the presence of the 1016G allele at 12 sites from nine countries, at frequencies ranging from 1 to 8% per sampling site (Fig. 1; Table 2; Additional file 1: Table S1). However, the sample size for some of the sites are low (Additional file 1: Table S1), and no detection of the 1016G mutation in these samples may imply low frequencies rather than absence in the whole population. Despite this limitation, we observed a spatial trend with the resistant allele being mostly detected in two clusters. The first cluster, hereafter called the “Western Cluster,” includes mainly Mediterranean coastal sites from Italy (Rome and Bari), France (Nice and Perpignan) and Malta (Luqa) but also sites in Spain (Basauri) and Switzerland (Basel). The second cluster, hereafter called the “Eastern cluster,” includes the easternmost sites on both sides of the Black sea from Bulgaria (Burgas), Turkey (Istanbul and Igneada) and Georgia (Batumi) as well as one site from Romania (Bucharest). Whether the observed clusters correspond to independent mutation or introduction events or reflect dispersal through migration and gene flow from the same source populations remains to be understood. Intriguingly, population genomic studies tracking the invasion history of *Ae. albopictus* in Europe revealed a pattern consistent with that of the 1016G *kdr* allele distribution. These studies showed high connectivity and close genetic relationship among Western European populations and identify Italian populations as possible bridgeheads for the invasion of other Western European countries [44–46]. In line with the present results, the same studies suggest that the populations from Eastern Europe originated from a different source population and that a major barrier to gene flow exists between Western European and Balkan clusters.

**Table 2:** Genotype and allele frequencies across European countries for wild-type (1016V) and mutated (1016G) alleles at position 1016 of the *vssc* gene in *Aedes albopictus* field populations sampled across Europe

Country	*N *sites	*N* specimens	Genotype frequency			1016G frequency
			VV	VG	GG	
Abkhazia (Georgia)	2	47	1.00	–	–	–
Albania	3	96	1.00	–	–	–
Bulgaria	2	107	0.935	0.065	–	0.033
Croatia	3	103	1.00	–	–	–
France	5	130	0.985	0.015	–	0.008
Georgia	1	49	0.980	0.020	–	0.010
Greece	5	283	1.00	–	–	–
Italy	2	71	0.845	0.155	–	0.077
Malta	1	50	0.960	0.040	–	0.020
Montenegro	3	126	1.00	–	–	–
Portugal	2	76	1.00	–	–	–
Romania	4	391	0.969	0.028	0.003	0.017
Russia	4	50	1.00	–	–	–
Serbia	7	274	1.00	–	–	–
Slovenia	1	40	1.00	–	–	–
Spain	14	384	0.995	0.005	–	0.003
Switzerland	6	65	0.954	0.046	–	0.023
Turkey	4	188	0.963	0.037	–	0.019
Total	69	2530	0.981	0.018	–	0.010

*G* Mutated 1016G allele, *V* wild-type 1016V allele

In this study, we focused on the detection of the 1016G mutation. However, other *kdr* mutations conferring pyrethroid resistance could contribute to a reduction in the susceptibility to pyrethroids and might even co-occur with the 1016G variant. In *Aedes aegypti*, a combination of the 1016G and 1534C alleles was found to have a strong synergistic effect, conferring increased resistance to pyrethroids in individuals carrying both mutations [32, 47]. In *Ae. albopictus*, specimens carrying both alleles have not been reported yet, despite both mutations in positions 1016 and 1534 circulate in populations from the native range in Vietnam [37] and China [34, 36]. Therefore, it would not come as a surprise to find co-occurrence of the two alleles also in Eastern Europe since the 1016G allele is found in countries neighboring Greece and mutation F1534C has been reported from Greece at frequencies up to 68% [48]. Moreover, the F1534C allele could likely be introduced into Italy by passive dispersal of *Ae. albopictus* specimens across the sea between Italy and Greece through extensive maritime traffic. Therefore, we highly recommend extending the insecticide resistance monitoring to include additional PCR diagnostics for alternative *kdr* alleles, including F1534C [49], and assessing the phenotype of possibly found multi-locus resistant populations.

## Conclusions

Genotyping of *kdr* mutations in major mosquito vector species such as *Ae. aegypti* and Afrotropical malaria vectors species has proven to be instrumental to trigger pyrethroid resistance management plans to slow down or reverse resistance spreading [41]. The present study represents the first effort to map the V1016G *kdr* mutation in *Ae. albopictus* on a continental scale in Europe.

On the one hand, results show that the very high frequencies previously reported from Italy are unparalleled in other European countries, consistently with a more extensive and/or protracted pyrethroid selective pressure in Italy. On the other hand, the presence of the 1016G allele in European populations both west and east of Italy represents a wake-up call for mosquito surveillance programs and highlights the need to include the monitoring of pyrethroid resistance in their activities. PCR genotyping of *kdr*-alleles represents a cost-effective and sensible tool to do this and, in case of detection of a sharp increase in frequencies, would allow timely implementation of policies to counteract inappropriate pyrethroid spraying for nuisance reduction and/or impose rotation of different pyrethroid-based adulticides for mosquito control. Notably, since insecticide use against agricultural pests is also known to represent an additional source of selective pressure for pyrethroid resistance in mosquitoes, rotation of different insecticidal compounds or enhanced integrated control measures in agriculture should also be considered [50]. This would prevent the risk of a reduced efficacy of emergency spraying in the case of an arbovirus outbreak.

Finally, the interactive map made available in this study includes all data so far available on 1016G allele distribution in Europe and will be updated with results from future genotyping studies on this and other *kdr* alleles. The map represents an easy tool for public health officers and private companies involved in mosquito control to assess the risk of pyrethroid resistance spreading in their regions in the early phases (i.e. when the frequency of V1016G or other *kdr* alleles is still low), thus opening the possibility to activate monitoring and management activities, instead of simply increasing pyrethroid concentrations, with inevitable harm to the environment and non-target species.

## Supplementary Information

**Additional file 1: Table S1.** Detailed genotyping results and samples collections/ processing information per sampling site.

## Data Availability

All data presented in the article are included in the article and its supplementary files; genotyping/sequencing data have been submitted to VectorBase.org (Project ID: VBP0000793).
